# Supplementary material for: Pentoxifylline Inhibits TNF-α/TGF-β1-Induced Epithelial-Mesenchymal Transition via Suppressing the NF-κB Pathway and SERPINE1 Expression in CaSki Cells
Source: Int J Mol Sci. 2023 Jun 24;24(13):10592. doi: 10.3390/ijms241310592 (PMC10342099; doi:10.3390/ijms241310592)
Supplement: Supplementary file 1 [file ijms-24-10592-s001.zip › ijms-2446102-supplementary.pdf]

Supplemental Figures

Pentoxifylline inhibits TNF-α/TGF-β1-induced epithelial-mesenchymal transition via suppressing the NF-κB pathway and *SERPINE1* expression in CaSki cells.

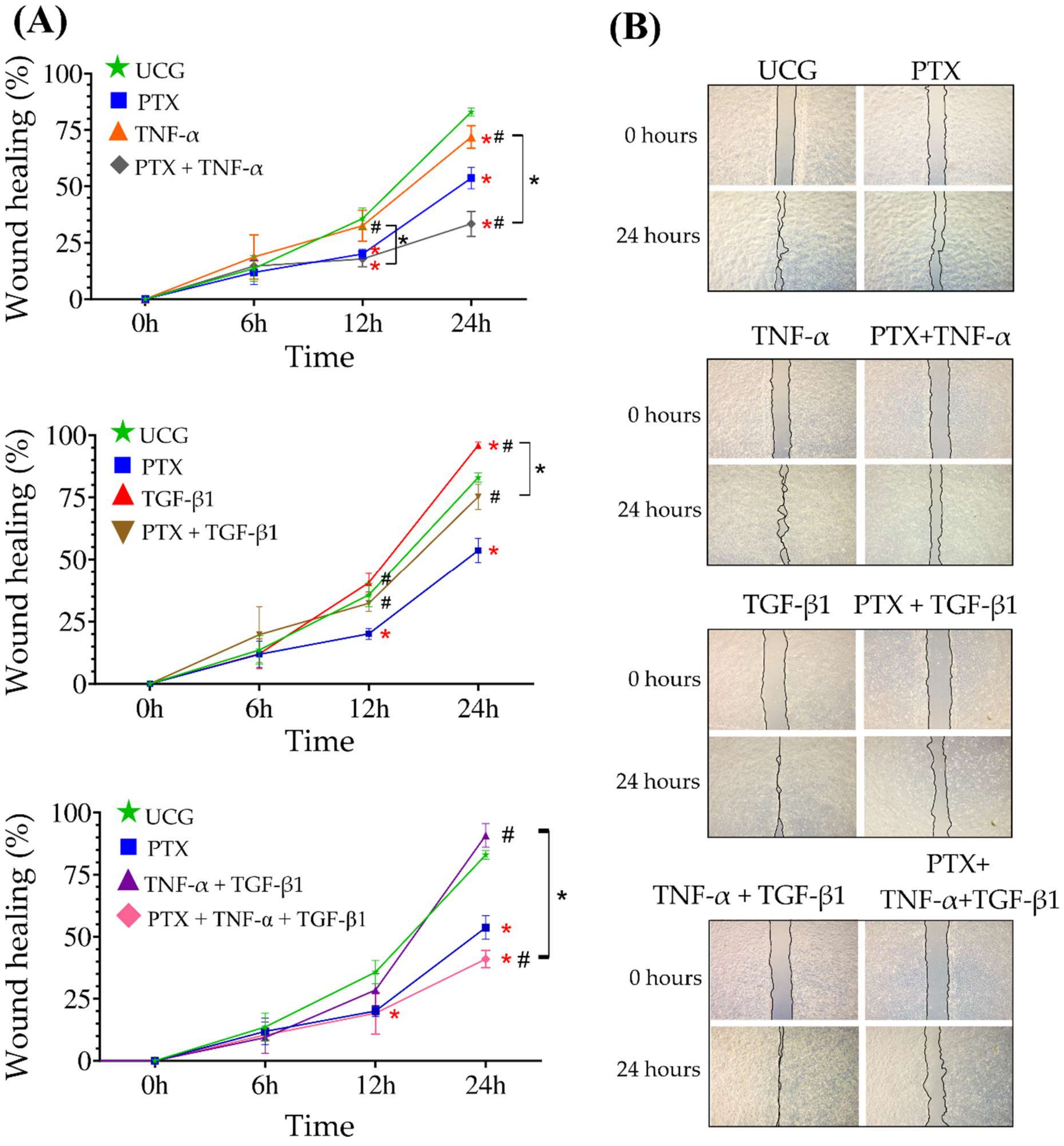

Supplementary Figure S1. Effect of PTX on the cell migration in cervical cancer CaSki cells by Wound healing assay: 1 × 10^5 cells/ml on a 6-well tissue culture plate, were treated with PTX, TNF-*α* and TGF-β1 alone or in combination for 5 days. After that, the scratch was realized. Results obtained from the time-lapse migration assay. (**A**) Wound healing difference was determined at 6, 12 and 24 h. (**B**) Representative images from a wound healing assay taken at 24 h. Data are represented as the mean ± SD of three separate experiments performed in triplicate. Statistical analysis: Mann–Whitney *U* test *$p < 0.05$ statistical significance in comparison with UCG, #$p < 0.05$ statistical significance in comparison with PTX groups, and *$p < 0.05$ statistical significance in the comparison between groups.

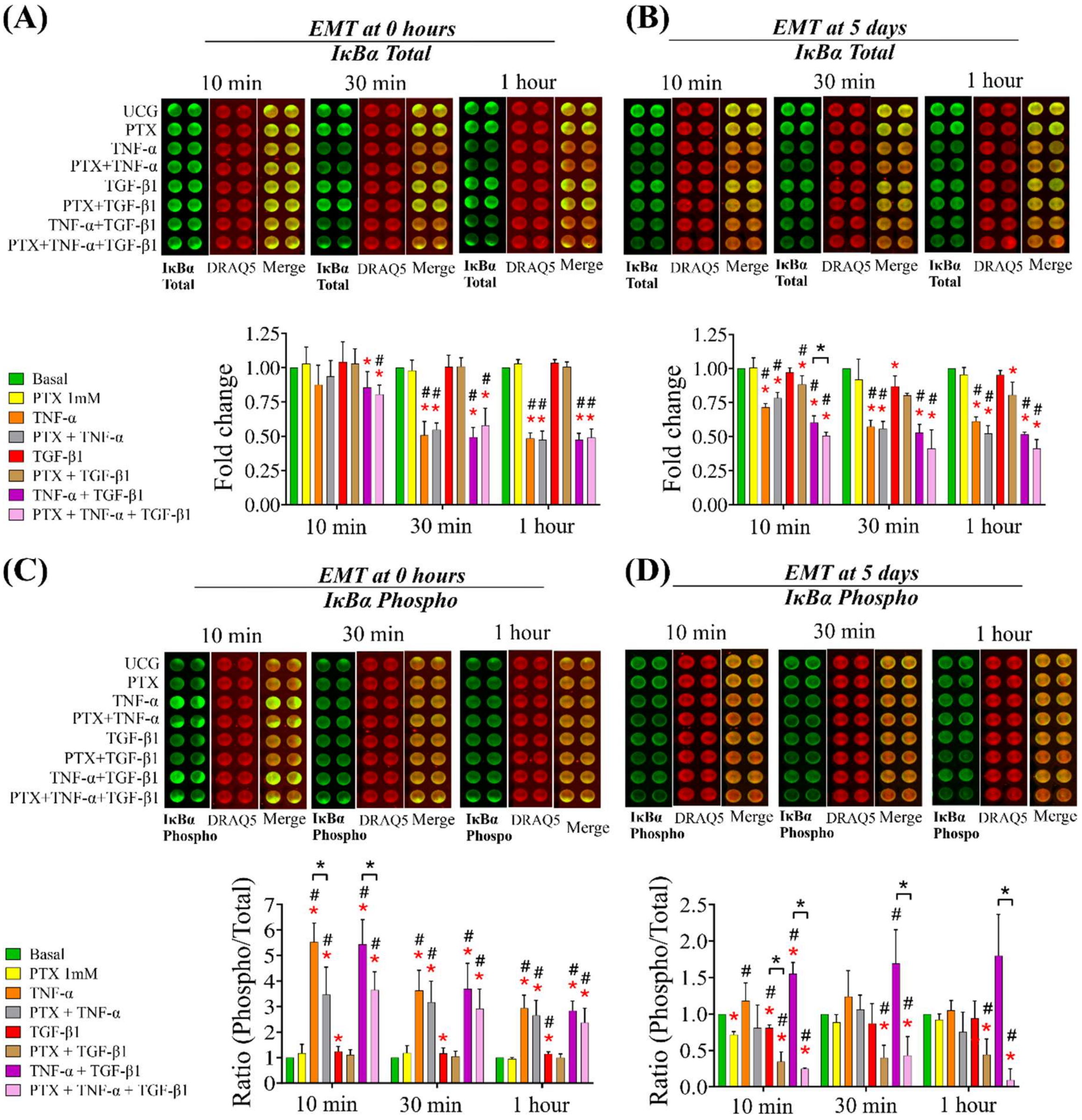

Supplementary Figure S2. Effect of PTX on p65 (S536) phosphorylation in epithelial and mesenchymal CaSki cells exposed to TNF-*α* or TGF-β1 or their combination by 1 h or 5 days, protein phosphorylation by ICW assay. ICW assay: CaSki epithelial cervical cancer cells (9x10^4 cells/wells) and mesenchymal-like CasKi cells (3.5x10^4 cells/wells) were grown in a 96-well optical black-walled transparent bottom. After 1 h treatment, CaSki cell monolayers were fixed in a methanol-acetone solution (3:1), permeabilized by Triton-X-100 in PBS. Antibodies, staining, and scanning as described in the material and methods. The proteins targeted were p65 and phospho-p65, examined in the epithelial and mesenchymal-like CasKi cells. (**A**) and (**B**) Representative images of an ICW plate. **(C)** and (**D**) Fold change in fluorescence of p65 total and phosphorylated at 10, 30 min, and 1 h. Data are represented as mean ± SD from three independent experiments performed in triplicate. Statistical analysis: Mann–Whitney *U* test. **p* < 0.05 statistical significance

in comparison with UCG, #$p < 0.05$ statistical significance in comparison with PTX groups, and *$p <$ 0.05 statistical significance in the comparison between groups.

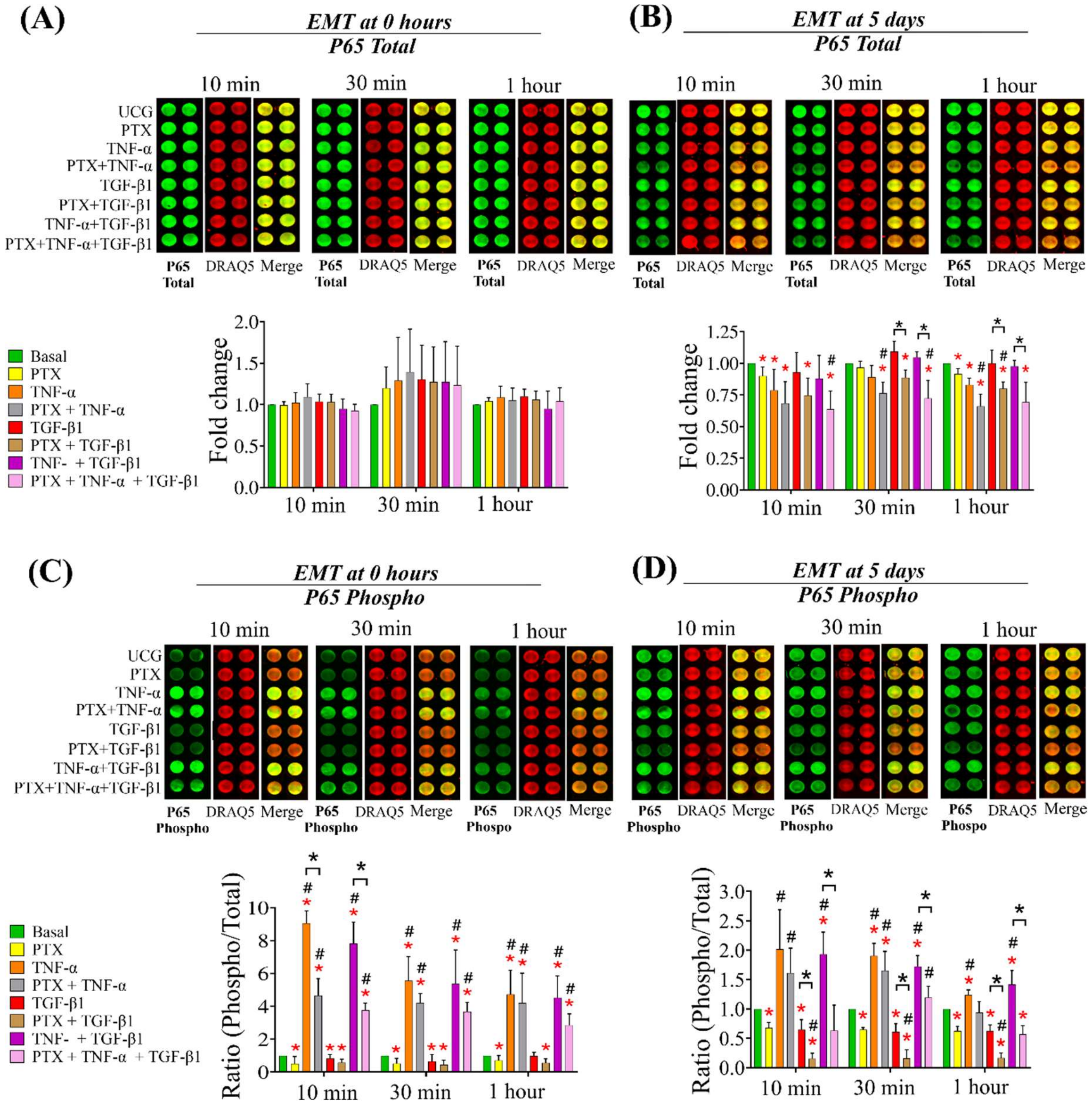

Supplementary Figure S3. Effect of PTX on IκBα total and IκBα (S-32) phosphorylation in epithelial and mesenchymal CaSki cells exposed to TNF-α or TGF-β1 or their combination by 1 h or 5 days, protein phosphorylation by ICW assay. ICW assay: CaSki epithelial cervical cancer cells (9x10^4 cells/wells) and mesenchymal-like CasKi cells (3.5x10^4 cells/wells) were grown in a 96-well optical, black-walled transparent bottom. After 1 h treatment, CaSki cell monolayers were fixed in a methanol-acetone solution (3:1), permeabilized by Triton-X-100 in PBS. Antibodies, staining, and scanning as described in the material and methods. The proteins targeted were IκBα total and IκBα and phospho-IκBα, examined in the epithelial and mesenchymal-like CasKi cells. (**A**) and (**B**) Representative images of an ICW plate. (**C**) and (**D**) Fold change in fluorescence of IκBα total and phosphorylated at 10, 30 min, and one h. Data are represented as mean ± SD from three

independent experiments performed in triplicate. Statistical analysis: Mann–Whitney *U* test. *$p < 0.05$ statistical significance in comparison with UCG, #$p < 0.05$ statistical significance in comparison with PTX groups, and *$p < 0.05$ statistical significance in the comparison between groups.
